# Depression and anxiety in different hypertension phenotypes: a cross-sectional study

**DOI:** 10.1186/s12991-022-00400-7

**Published:** 2022-06-27

**Authors:** Zsófia Nemcsik-Bencze, Beáta Kőrösi, Helga Gyöngyösi, Dóra Batta, Andrea László, Péter Torzsa, Illés Kovács, Zoltán Rihmer, Xénia Gonda, János Nemcsik

**Affiliations:** 1 Department of Neuroradiology, Semmelweis University, Budapest, Hungary; 2 Department of Family Medicine, Semmelweis University, Budapest, Hungary; 3 Norisana – MVZ Rosenau, Nuremberg, Germany; 4 Department of Ophthalmology, Semmelweis University, Budapest, Hungary; 5 Department of Ophthalmology, Weill Cornell Medical College, New York City, NY USA; 6 Department of Psychiatry and Psychotherapy, Semmelweis University, Budapest, Hungary; 7 Department of Pharmacodynamics, Semmelweis University, Budapest, Hungary; 8 Neurochemistry Research Group, MTA-SE, Budapest, Hungary; 9 Health Service of Zugló (ZESZ), Budapest, Hungary

**Keywords:** Depression, Anxiety, Resistant hypertension, White-coat hypertension

## Abstract

**Background:**

Hypertension is a major risk factor of cardiovascular mortality. Mood disorders represent a growing public health problem worldwide. A complex relationship is present between mood disorders and cardiovascular diseases. However, less data is available about the level of depression and anxiety in different hypertension phenotypes. The aim of our study was to evaluate psychometric parameters in healthy controls (Cont), in patients with white-coat hypertension (WhHT), with chronic, non-resistant hypertension (non-ResHT), and with chronic, treatment-resistant hypertension (ResHT).

**Methods:**

In a cross-sectional study setup 363 patients were included with the following distribution: 82 Cont, 44 WhHT, 200 non-ResHT and 37 ResHT. The patients completed the Beck Depression Inventory (BDI) and the Hamilton Anxiety Scale (HAM-A).

**Results:**

BDI points were higher in WhHT (7 (3–11)) and ResHT (6 (3–11.5)) compared with Cont (3 (1–6), *p* < 0.05). Similarly, HAM-A points were higher in WhHT (8 (5–15)) and ResHT (10.5 (5.25–18.75)) compared with Cont (4 (1–7), *p* < 0.05) and also compared with non-ResHT (5 (2–10), *p* < 0.05). ResHT was independently associated with HAM-A scale equal or above 3 points (Beta = 3.804, 95%CI 1.204–12.015). WhHT was independently associated with HAM-A scale equal or above 2 points (Beta = 7.701, 95%CI 1.165–18.973) and BDI scale equal or above 5 points (Beta = 2.888, 95%CI 1.170–7.126).

**Conclusions:**

Our results suggest psychopathological similarities between white-coat hypertension and resistant hypertension. As recently it was demonstrated that white-coat hypertension is not a benign condition, our findings can have relevance for future interventional purposes to improve the outcome of these patients.

## Background

Hypertension is considered as the leading cause of cardiovascular (CV) mortality and based on American data, it is in the second place in the list of the preventable causes of all-cause mortality as well [1, 2]. Mood disorders such as depression and anxiety represent a growing public health problem and their association with adverse CV outcome is well-established [3–5].

The association between hypertension, depression and anxiety are controversially discussed in diverse studies [6, 7]. The background of this observation can be based on the differences of the studied patient populations, the application of different psychometric measures, the heterogenous pathophysiological background of hypertension or the presence of different hypertension phenotypes in the cohorts. Such phenotypes are white-coat hypertension (WhHT), masked hypertension, chronic, treatment-resistant hypertension (ResHT) and chronic, non-resistant hypertension (non-ResHT). In WhHT patients blood pressure is increased in office, but normal during out-of office measurements. In masked hypertension home results are elevated, while in the office, blood pressure is normal [8]. In ResHT blood pressure is above 140/90 mmHg in the office in spite of the use of 3 antihypertensive drugs of different classes including a diuretic. ResHT also includes patients whose blood pressure is controlled with the use of more than 3 medications [9]. It was demonstrated that both controlled and uncontrolled ResHT are accompanied with higher CV risk compared with non-ResHT [10].

Anxiety was found to be associated with white-coat effect during blood pressure measurement [11, 12], while resistant hypertension was also associated with anxiety [13]. Recently we described similarities in affective temperament patterns between WhHT and ResHT [14]. However, until now the level of depression and anxiety was not evaluated in a cohort of individuals with different hypertension phenotypes.

The aim of our study was to measure depression and anxiety in healthy controls, in untreated subjects with white-coat effect and in chronic hypertension with or without the presence of resistant hypertension. Based on our recent findings we hypothesized similarities between subjects with white-coat and resistant hypertension.

Methods.

Our present results are part of an additional analysis of a previously published cohort [14], including the results of the depression and anxiety questionnaires.

### Patients

In the setup of a cross-sectional study, altogether 363 Caucasian patients were involved between August 2012 and January 2019, in three primary care practices in Budapest, Hungary. Four categories of the enrolled subjects were defined: healthy controls (Cont, *n* = 82); white-coat hypertensive patients (WhHT, *n* = 44); chronic, non-resistant hypertensive patients (non-ResHT, *n* = 200); and patients with chronic, resistant hypertension (ResHT, *n* = 37). The diagnosis of resistant hypertension was always confirmed by ambulatory blood pressure monitoring (ABPM) or by home blood pressure monitoring (HBPM). The definition of non-resistant hypertension required the following criteria: chronic (the onset is longer than 3 months), treated (with the maximum use of 3 antihypertensive agents) and controlled hypertension.

As in this cohort carotid–femoral pulse wave velocity was also measured (data are not shown in the present publication), patients with atrial fibrillation were excluded from the study. Other exclusion criteria were treated depression, anxiety, or other psychiatric conditions (bipolar disorder, schizophrenia) and also dementia to avoid mistakes or misunderstanding of the questionnaires. The chronic, moderate use of alprazolam (< 0.5 mg/day) was not an exclusion criterion when it was added to the hypertension therapy protocol by other specialists, without the diagnosis of anxiety.

Subjects were recruited into the study during the screening visit when blood pressure was measured with a validated oscillometric device (Omron M3) and written informed consent was collected. At the end of the screening visit an autoquestionnaire was handed out to the subjects including a questionnaire for the evaluation of family and personal history and also for depression. The autoquestionnaires were collected from the patients in the day of the clinical measurements.

After the screening visit within the maximum of a 2-week period an appointment was scheduled for 7.00. a.m. for repeated office and/or ambulatory blood pressure measurement and also for blood sampling. Finally the evaluation of anxiety was completed in the form of an interview with the examiner.

### Evaluation of depression and anxiety

The *Beck Depression Inventory* (BDI) was used to evaluate the severity of depression. It is one of the most widely used instruments including a 21-question multiple-choice self-report questionnaire with ratings on a four-point scale, where a higher score correlates with more severe depression [15].

The *Hamilton Anxiety Scale* (HAM-A) was used to study the severity of anxiety. During the evaluation the examiner reports the subject. Its scale consists 14 items, each item is scored on a scale of 0 (not present) to 4 (severe anxiety) [16].

### Office and ambulatory blood pressure measurement

Before the clinical measurements overnight fasting, refrain from smoking and drinking caffeine-containing beverages were required, but patients were asked to take their usual antihypertensive medication. In sitting position after 5 min of rest, two brachial blood pressure measurements were taken in 1-min interval on each arm with an oscillometric device (Omron M3, validated). In the detailed analysis the mean value of the higher side of arms was further used as brachial systolic (SBP) and diastolic (DBP) blood pressures and heart rate. Pulse pressure (PP) was also calculated as SBP minus DBP. Next, untreated subjects, who had elevated office blood pressure during the screening visit, were fitted with a 24-h ABPM device (Mobil-O-Graph, I.E.M. GmbH, Germany). The cuff was placed on the left arm. Finally venipuncture was performed on the right arm for blood sampling. The 24-h ABPM and blood test results were discussed with the patient on the following day. In case of treated hypertensive patients with increased office blood pressure, where the diagnosis of ResHT was considered, it was confirmed within 2 weeks with HBPM or with ABPM.

### Statistical analysis

Descriptive data are expressed as mean ± standard deviation or median with interquartile ranges. Kolmogorov–Smirnov test was used test the normality of continuous parameters. ANOVA was applied for normally distributed parameters to compare differences between the four groups (Cont, WhHT, non-ResHT, ResHT). Tukey's test was used for post-hoc analysis. Kruskal–Wallis test was applied to compare non-normally distributed parameters.

To study the association of depression and anxiety with ResHT, chronic hypertensive patient groups (non-ResHT plus ResHT) were dichotomized based on the 75% quartile of the depression and anxiety scores of the patients. Next, with binary regression analysis, the association with ResHT of the patient groups reaching different BDI and HAM-A scores was studied with the adjustment for traditional CV risk factors, such as age, sex, smoking, diabetes, body mass index and total cholesterol. Finally, in the merged group of Cont plus WhHT, with binary regression analysis the association with WhHT of the patients reaching different BDI and HAM-A points was also studied with the adjustment for sex, smoking and BMI.

In all analyses *p* < 0.05 was considered as the border of significance. SPSS 22.0 for Windows was used throughout the calculations.

## Results

Demographic parameters and comorbidities, results of blood sampling, current medication and the number of the used antihypertensive medications are summarized in Table 1.

**Table 1:** Baseline characteristics of the study participants

	Cont	WhHT	non-ResHT	ResHT
N (male/female)	82 (31:51)	44 (22:22)	200 (91:109)	37 (14:23)
Age (years)	49.61 ± 18.28	44.12 ± 13.36	***59.44* ± *13.24***	***65.55* ± *10.22***
Duration of hypertension (years)	−	−	9.40 ± 9.11	17.44 ± 13.22
Diabetes [*n* (%)]	−	3 (3.7%)	32 (16%)	16 (43%)
CV disease [*n* (%)]	−	−	18 (9%)	12 (32%)
Current smoker [*n* (%)]	12 (15%)	8 (18%)	38 (19%)	7 (19%)
BMI (kg/m^2^)	24.35 ± 3.65	***27.70* ± *5.62***	***28.80* ± *4.62***	***30.50* ± *3.45***
Blood glucose (mmol/l)	4.90 (4.60–5.51)	5.15 (4.75–5.40)	5.49 (5.00–6.32)	6.28 (5.26–7.50)
GFR–EPI (ml/min/1.73m^2^)	79.31 ± 32.40	***93.31* ± *30.09***	81.69 ± 18.37	72.35 ± 24.24
Uric acid (µmol/l)	295.00 ± 72.97	307.61 ± 68.92	***327.86* ± *81.82***	***351.59* ± *101.05***
Total cholesterol (mmol/l)	5.41 ± 1.10	5.54 ± 1.38	5.41 ± 1.15	***4.76* ± *1.33***
LDL (mmol/l)	3.30 ± 0.97	3.56 ± 1.24	3.34 ± 1.03	***2.73* ± *1.24***
HDL (mmol/l)	1.64 ± 0.36	1.47 ± 0.37	***1.38* ± *0.38***	***1.26* ± *0.32***
Triglyceride (mmol/l)	0.96 (0.69–1.30)	1.17 (0.87–1.57)	***1.48 (1.08–2.05)***	***1.53 (1.10–2.47)***
Medication [*n* (%)]				
ACE-inhibitor	−	−	118 (59%)	24 (64.9%)
ARB [*n* (%)]	−	−	29 (14.5%)	12 (32.4%)
Calcium channel blocker	−	−	75 (37.5%)	28 (75.6%)
Beta-blocker	−	−	89 (44.5%)	27 (72.9%)
Diuretic	−	−	23 (11.5%)	33 (89.2%)
Alfa-adrenergic receptor blocker	−	−	22 (11%)	19 (51.3%)
Centrally acting agents	−	−	1 (0.5%)	−
Direct acting vasodilators	−	−	1 (0.5%)	5 (13.5%)
Antiplatelet drug	1 (1.2%)	−	42 (21%)	12 (32.4%)
Statin	6 (7.3%)	1 (2.3%)	50 (25%)	12 (32.4%)
Fibrate	−	−	7 (3.5%)	5 (13.5%)
Alprazolam	1 (1.2%)	−	18 (9%)	2 (5.4%)
Number of antihypertensive medications				
0	82 (100%)	44 (100%)	−	−
1	−	−	85 (42.5%)	−
2	−	−	72 (36%)	−
3	−	−	43 (21.5%)	16 (43.2%)
4	−	−	−	9 (24.3%)
5	−	−	−	9 (24.3%)
6	−	−	−	2 (5.4%)
7	−	−	−	1 (2.7%)

Data are presented as mean ± standard deviation or median (interquartile ranges)

*Cont*: healthy controls, *WhHT*: patients with white-coat hypertension, *non-ResHT*: chronic, non-resistant hypertensive patients, *ResHT*: chronic, resistant hypertensive patients, *CV* diseases: cardiovascular diseases, *BMI*: body mass index, *GFR*–*EPI*: glomerular filtration rate assessed by the chronic kidney disease epidemiology collaboration glomerular filtration rate equation, *LDL*: low-density lipoprotein, *HDL*: high-density lipoprotein, *ACE*: Aangiotensin converting enzyme, *ARB*: angiotensin II receptor blocker

Categorical parameters are presented as *n* (%). Significant differences compared with Cont are signed as bold and italic characters

Compared with Cont, non-ResHT and ResHT patients had elevated age, higher BMI, blood glucose, uric acid and triglyceride levels, and lower HDL cholesterol. ResHT patients had decreased total and LDL cholesterol levels compared with Cont probably as a consequence of the administration of statins. BMI and eGFR were higher in WhHT compared with Cont.

Hemodynamic parameters and the results of BDI and HAM-A questionnaires are summarized in Table 2.

**Table 2:** Hemodynamic parameters, depression and anxiety scores of the different groups of patients

	Cont	WhHT	non-ResHT	ResHT
Hemodyn. parameters				
Hearth rate (1/min)	72.1 ± 10.7	77.61 ± 11.08	74.1 ± 20.58	73.87 ± 13.53
SBP (mmHg)	121.36 ± 11.40	***136.45* ± *12.24***	***131.40* ± *11.9***	***143.96* ± *20.86 #***
DBP (mmHg)	73.18 ± 7.83	***82.91* ± *6.96 #***	76.02 ± 10.21	***79.17* ± *11.19***
PP (mmHg)	47.74 ± 8.94	***47.68* ± *9.46 #***	***53.85* ± *11.30***	***58.02* ± *14.24***
BDI	3.0 (1.0–6.0)	7.0 (3.0–11.0)*	4 (2.0–8.0)	6.0 (3.0–11.5)*
HAM-A	4.0 (1.0–7.0)	8.0 (5.0–15.0)*#	5.0 (2.0–10.0)	10.5 (5.25–18.75)*#

Data are presented as mean ± standard deviation or median (interquartile ranges)

*Cont*: healthy controls, *WhHT*: patients with white-coat hypertension, *non-ResHT*: chronic, non-resistant hypertensive patients, *ResHT*: chronic, resistant hypertensive patients, *SBP*: brachial systolic blood pressure, *DBP*: brachial diastolic blood pressure, *PP*: brachial pulse pressure, *BDI*: Beck Depression Inventory, *HAM-A*: Hamilton Anxiety Scale

Categorical parameters are presented as *n* (%). Significant differences compared with Cont are signed as bold and italic characters. Significant differences compared with non-ResHT are signed as bold characters with #

**Fig. 1:**
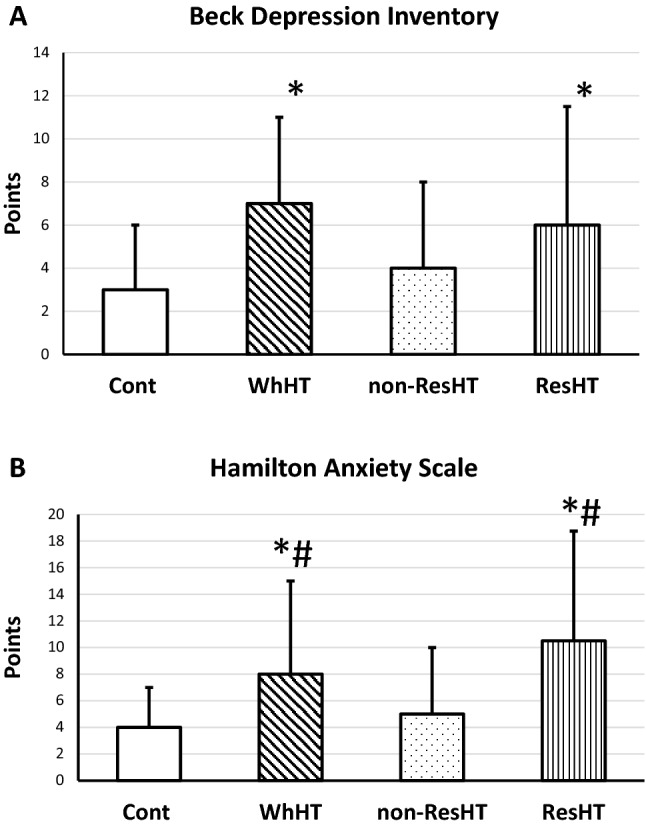
Differences between healthy controls (Cont), white-coat hypertensive (WhHT), chronic, non-resistant hypertensive (non-ResHT) and chronic, resistant hypertensive patients (ResHT) in the degree of depression (**A**) and anxiety (**B**). *p < 0.05 compared with Cont; #*p* < 0.05 compared with non-ResHT

Compared with Cont SBP was higher in WhHT, non-ResHT and ResHT groups. SBP in ResHT was even higher than in non-ResHT. Compared with Cont, DBP was higher only in WhHT and ResHT. BDI points of WhHT and ResHT groups were significantly higher compared with Cont (*p* < 0.05). Similarly, compared with Cont, HAM-A points of WhHT and ResHT were also higher, but these two groups of patients had higher HAM-A points compared with non-ResHT as well (*p* < 0.05). Figure 1 also demonstrates the differences in the depression and anxiety points between the different study groups.

Table 3 demonstrates the independent association of depression with white-coat hypertension.

**Table 3:** Association of depression evaluated with the presence of white-coat hypertension in healthy controls and white-coat hypertensive patients (*n* = 126). Binary regression analysis, adjusted for multiple confounders

	*B*	Beta	95% CI		*p*
			Lower	Upper	
Age	−0.012	0.989	0.954	1.024	0.525
Sex	−0.194	0.824	0.320	2.124	0.688
Smoking	0.332	1.394	0.429	4.525	0.581
BMI	0.146	1.157	1.025	1.307	0.019
Total cholesterol	0.168	1.183	0.803	1.742	0.396
GFR–EPI	0.026	1.026	0.995	1.059	0.100
BDI min. 5 points	1.060	2.888	1.170	7.126	***0.021***

95% CI, lower–upper: 95% confidence interval, lower and upper values; *p* < 0.05 are signed with bold and italic characters. *BMI*: body mass index; *GFR*–*EPI*: glomerular filtration rate assessed by the chronic kidney disease epidemiology collaboration glomerular filtration rate equation; *BDI*: Beck Depression Inventory

After the adjustment of multiple variables white-coat hypertension was associated with BDI scale equal or above 5 (Beta = 2.888, 95% CI 1.170–7.126) points.

Table 4 demonstrates the independent association of anxiety with white-coat and resistant hypertension.

**Table 4:** Association of anxiety with the presence of white-coat hypertension in healthy controls and white-coat hypertensive patients (**A**, *n* = 126) and with the presence of resistant hypertension in chronic hypertensive patients (**B**, *n* = 237). Binary regression analysis, adjusted for multiple confounders

(A)					
	*B*	Beta	95% CI		*p*
			Lower	Upper	
Age	−0.014	0.986	0.951	1.022	0.444
Sex	−0.203	0.817	0.320	2.085	0.672
Smoking	0.198	1.22	0.367	4.053	0.746
BMI	0.136	1.145	1.012	1.295	0.031
Total cholesterol	0.160	1.174	0.787	1.751	0.431
GFR–EPI	0.027	1.028	0.995	1.061	0.095
HAM-A min. 2 points	1.548	4.701	1.165	18.973	***0.030***

(B)					
Age	0.017	1.018	0.975	1.062	0.425
Sex	−0.058	0.944	0.405	2.197	0.893
Diabetes	0.855	2.352	0.952	5.809	0.064
Smoking	0.598	1.818	0.626	5.280	0.272
BMI	0.101	1.106	1.011	1.210	0.027
Total cholesterol	−0.281	0.755	0.516	1.106	0.149
GFR–EPI	−0.013	0.987	0.965	1.010	0.252
HAM-A min. 3 points	1.336	3.804	1.204	12.015	***0.023***

95% CI, lower–upper: 95% confidence interval, lower and upper values; *p* < 0.05 are signed with bold and italic characters

*BMI*: body mass index, *GFR*–*EPI*: glomerular filtration rate assessed by the chronic kidney disease epidemiology collaboration glomerular filtration rate equation, *HAM-A*: Hamilton Anxiety Scale

After the adjustment of multiple variables white-coat hypertension was associated with HAM-A scale equal or above 2 points (Beta = 4.701, 95% CI 1.165–18.973, Table 4A), while resistant hypertension was associated with HAM-A scale equal or above 3 points (Beta = 3.804, 95% CI 1.204–12.015, Table 4B).

## Discussion

In our study we demonstrated that the level of depression and anxiety can vary in different hypertension phenotypes. Patients with white-coat and resistant hypertension scored higher points compared with healthy controls in BDI scale, and also compared with non-resistant hypertensive patients in HAM-A scale. In addition, almost similar threshold limits were found in respect of HAM-A scale with the independent association of white-coat and resistant hypertension.

Our results are in line with our recent findings on the same cohort, where cyclothymic affective temperament points were similarly higher in white-coat and resistant hypertension compared with healthy controls and cyclothymic affective temperament points equal or above 6 were independently associated both with white-coat and resistant hypertension [14]. Therefore, in addition to cyclothymic affective temperament, another psychopathological similarities, namely, the level of depression and anxiety, are also present between white-coat and resistant hypertensive patients. As in our study patients with untreated white-coat hypertension were much younger compared with the resistant hypertensive ones, these results suggest that those white-coat hypertensive patients who progress to sustained hypertension, are prone to develop resistant hypertension, which is a novel hypothesis and requires long-lasting prospective studies to confirm. However, we suppose that in the present stage our results are enough to encourage clinicians to pay more attention for the psychopathological features of white-coat hypertensive patients, which is particularly important in the light of a very recent study. Until now untreated white-coat hypertension was supposed to be a benign phenomenon, but the study of Mancia et al. on PAMELA cohort with the median follow-up of 29 years demonstrated that white-coat hypertension with or without organ damage is associated with elevated CV and all-cause mortality risk compared with normotension [17]. These results will probably modify the recommendations about the clinical management of white-coat hypertension and based on the results of our present study as target of intervention, depression and anxiety might be considered.

However, it is the first study which evaluated the level of depression and anxiety in different hypertension phenotypes in one cohort, in the literature some data are already available about the similarities between white-coat and resistant hypertension. Anxiety has a pathophysiological role in both conditions as it was found to be associated with white-coat effect [12] and was also more pronounced in resistant hypertension compared with uncontrolled, but not resistant hypertensive patients [13]. Depression and stress were also found to be associated both with white-coat and resistant hypertension [18–20]. Our results are in line with these previous studies and confirm the importance of the evaluation of mood disorders in hypertension, especially as proper medical intervention of mood disorders might improve hypertensive conditions as well [21].

A limitation of our study was that although standardized autoquestionnaires were used and patients with dementia were excluded, the possibility of misinterpretations or mistakes during the completion of the questionnaires could not have been totally excluded. Next, the cross-sectional design of the study limits us to make causal inferences. Furthermore, as all of participants were from Caucasian race it limits the generalizability of our results for other races. Finally, as ABPM was not performed in those patients or healthy participants who had normal office blood pressure values and did not report elevated values during home blood pressure monitoring, we were unable to diagnose masked hypertension and to analyze the level of depression and anxiety of this hypertension phenotype.

## Conclusions

Psychopathological similarities are present between white-coat and resistant hypertensive patients. In light of the recent evidence as white-coat hypertension is not a harmless condition, our findings can have relevance for future interventional purposes to improve the outcome of these patients.

## Abbreviations

ACE-inhibitor: Angiotensin-converting enzyme inhibitor
ARB: Angiotensin II receptor blocker
BDI: Beck Depression Inventory
BMI: Body mass index
CV: Cardiovascular
CKD–EPI GFR: Glomerular filtration rate assessed by the chronic kidney disease epidemiology collaboration glomerular filtration rate equation
DBP: Brachial diastolic blood pressure
HAM-A: Hamilton Anxiety Scale
HDL: High-density lipoprotein
PP: Brachial pulse pressure
SBP: Brachial systolic blood pressure
TEMPS-A: The Temperament Evaluation of Memphis Pisa, Paris and San Diego questionnaire

## Declarations

### Availability of data and materials

The data presented in this study are available on request from the corresponding author. The data are not publicly available due to reasons pertaining to patient privacy.
